# Unlocking safety: a mathematical model and selective extraction for Δ9-THC removal from hemp seed husks

**DOI:** 10.1186/s42238-025-00349-w

**Published:** 2025-11-17

**Authors:** Namsoo P. Kim, Abhilash Aditiya, Too Jae Min

**Affiliations:** 1 VIZO Investment, INC., Washington DC, USA; 2 Dept. of Pharmacognosy and Pharmaceutical Botany, Chulalongkorn University, Bangkok, Thailand; 3 College of Engineering, University of Texas , El Paso, TX USA; 4 Dept. of Anesthesiology and Pain Medicine, Korea University Ansan Hospital, Ansan , Gyeonggi-do Korea

**Keywords:** Δ9-THC, Hemp seed, Selective THC removal, PK-16 analysis

## Abstract

Among the myriad components found in marijuana, cannabis, and hemp, cannabidiol (CBD), delta 9-tetrahydrocannabinol (Δ9-THC), cannabinol (CBN), cannabigerol (CBG), and other cannabinoids have long been utilized. However, controversies persist regarding the addictive and hallucinogenic effects of Δ9-THC, CBN, and hexahydrocannabihexol (HHCH) in various countries, contrasting with the positive attributes associated with CBD and CBG. Ongoing debates and sudden regulatory shifts pose challenges for the medical and industrial utilization of hemp, despite the UN’s favorable stance persisting in many countries. Despite the widespread use of hemp seeds or oils in the food and medical sectors, many countries also negatively evaluate hemp product use due to the ambiguity of the Δ9-THC content analysis methods and corresponding standards. These difficulties arise from concerns regarding economic viability, regulatory restrictions on Δ9-THC levels, and the lack of stable processing and analysis techniques. To address these issues, a mathematical model based on hemp seed density was initially proposed. This model aims to analyze husk thickness and Δ9-THC concentration, thereby improving the stability and production efficiency of hemp seeds and making their utilization economically feasible. By expanding upon the mechanical peeling model, chemical extraction was conducted to control cannabinoids in hemp seeds using oil roasting and aqueous solutions such as ethanol based on the difference in the solubility of cannabinoids in hydrophilic and hydrophobic solutions. Different techniques were examined within a mathematical framework. THC removal was confirmed through repeated experiments and analysis of the residual THC content. PK-16 analysis revealed that the husk components contained 300 mg/kg THC, and the nut kernels contained 2 mg/kg THC. THC was selectively removed without physically eliminating husks, establishing a simplified, efficient, and economically viable method for future hemp seed oil production and analysis.

## Background 

### The status of hemp

Edible and medical cannabis oils are facing difficulties due to scientific regulations and economic feasibility. The contemporary environmental status of hemp presents challenges regarding cannabis, particularly focusing on addiction and hallucination due to the concentrations of CBD and Δ9-THC. While substantial evidence supports both compounds, CBD’s safety is widely acknowledged without any country openly refuting it. In contrast, the status of Δ9-THC is less clear, leading to uncertainties regarding its permissible extent of use. Even in jurisdictions with lenient policies, potential risks are recognized(Rapin et al. 2021). This prompts challenges such as high costs and storage difficulties, especially during the removal of Δ9-THC-laden husks, where approximately one-third of the total product is discarded during drying and mechanical peeling processes in Asia(Kim 2023b). Despite these challenges, the roasting process for component transformation extraction inevitably alters the composition. These processes are characterized by prohibitively high costs, inherent instability, lack of quality control for the product, and the ideological contention that THC-safe certified hemp may render the plant obsolete, potentially distorting its millennium-old narrative of survival(Vincent Rajkumar 2020).

The primary objective of this paper is to address the most pressing and pragmatic issues at hand. Theoretically, if Δ9-THC is predominantly concentrated in husks rather than in nut kernels, utilizing principles of engineering diffusion theory, it may be possible to structurally remove externally located cannabinoids based on their spatial distribution. The medicinal application of hemp in Asia is transforming, shifting from being a historical tool of legislation to becoming a source of political issues. Oscillating between total prohibition and full legalization obscures the truth, leading to a situation where farmers, producers, and users are all viewed through the lens of potential criminality, depending on one’s perspective. Thailand, which recently legalized cannabis for recreational purposes, announced that it would be prohibited until December 2024(Reuters 2024).

The safe delivery of economically viable CBD on a global scale, whether THC-free or THC-safe, should not be restricted due to these longstanding political and economic reasons, thereby preventing the potential for traditional usage by individuals in a nation. As of 2024, Korean laws strictly regulate THC content, prohibiting the use of hemp buds, flowers, and leaves and imposing rigorous scrutiny on hemp seeds. This poses significant challenges for economic viability and product stability. To address this issue, the author observed hemp oil production at a farm. During hemp oil production, nut kernels are created primarily for compression. In a recent instance in Andong, South Korea, processing 11 kg of hemp seeds yielded approximately 4.5 kg of nut kernels(Kim 2023a, b, c).

A significant portion of the outer husk, comprising 40–60% of the original weight, is discarded during husk removal, emphasizing the need for an economically viable method. Without such a method, traditional processes result in the excessive removal of nut kernels. Therefore, a mathematical model for extraction and simulation is essential to expand the commercial viability of hemp in medical and industrial markets. In the case of South Korea, efforts were made in 2020 to designate Andong as a special zone for cannabis GMP-level production, considering its specialization in agriculture. However, due to a lack of expertise, research findings have failed to translate into commercialization(류석우 2023).

### Insights from traditional medical texts

Historically, cannabis seeds have been extensively used in the Oriental literature for medicinal purposes, with a focus on their low cannabinoid content compared to that of other plant parts, such as buds, flowers, and leaves. Hempseed, which is derived from the *Cannabis sativa* plant, has been used medicinally for centuries across various cultures. Traditional medical texts contain valuable knowledge about their therapeutic properties. Hempseed has a rich history of medicinal use dating back to ancient civilizations such as China, India, Egypt, Israel, Japan, Thailand, and Korea. However, in the East, the pharmacological components, extraction methods, and clinical applications of hemp for health foods and medical purposes are increasingly being patented based on recent Western analytical technologies(Kim 2023a).

In the Korean traditional medical book DongeuiBogam (동의보감), there is a passage in the “method to see the ghost” (見鬼方) section that discusses methods for seeing ghosts. The passage reads: “要見鬼者 取生麻子 石菖蒲 鬼臼等分 爲末 蜜丸彈子大 每朝向日服一丸 服滿百日 卽見鬼.” This translates to “Those who want to see ghosts should grind equal amounts of hemp seeds, Acorus gramineus (石菖蒲), and Dysosmae Rhizoma (鬼臼) into powder and make pills. Take one pill every morning facing the sun, and after taking it for 100 days, you will be able to see ghosts.” Interpreting the original text in the current context presents challenges. It should be understood that the mention of hemp or cannabis seeds in the original manuscript likely refers to the psychoactive effects of THC rather than pain relief. This paper proposes a novel method to extract both lipophilic and hydrophilic components at approximately 100 °C, aiming to enhance extraction efficiency from husks(Heo 2012).

Inspired by traditional methods described in ancient texts such as Shengji Zonglu (聖濟總錄), this method selectively extracts Δ9-THC and CBD from hemp husk without removing it entirely, optimizing efficiency and compliance with regulations. The treatment of alopecia using oil extracted from roasted hemp seeds has a historical background. “尸咽痛癢” refers to a condition of a sore and itchy throat. “麻子燒脂,酒調一錢 服之” refers to the consumption of one unit of oil extracted from roasted hemp seeds mixed with alcohol. This method of extracting oil from roasted hemp seeds and using it to treat a condition characterized by a sore and itchy throat shows traces of treating a kind of parasite (probably referring to a throat ailment) during that time. It is noteworthy that historical records mention various methods of roasting, brewing, or extracting oil from hemp and using it for medicinal purposes(Zhao Ji 1117).

Similarly, in Ayurveda (आयुर्वेद), an ancient medical system in India, hempseed, known as भङ्ग (Bhanga), has analgesic, anti-inflammatory, and anxiolytic properties. In ancient Egypt, hempseed was revered for its nutritional and therapeutic benefits, as evidenced by its depiction in the Ebers Papyrus. Traditional medical texts prescribe hempseed for conditions such as rheumatism, constipation, and menstrual disorders. Ayurvedic texts recommend it for managing pain, anxiety, and gastrointestinal ailments(Pandey et al. 2013). Additionally, hemp seed oil is used topically for skin conditions and as a rejuvenating tonic in both systems of medicine. The nutritional value of hemp seed is also highlighted, emphasizing its role in nourishing the body and promoting overall well-being. Through experiments and mathematical modeling, this paper demonstrated effective Δ9-THC extraction while minimizing the costs associated with husk removal. This technology has potential applications in selective chemical removal techniques based on extended diffusion and extraction. In Thailand, the process of preparing crude cannabis drugs before making medicine involves the use of “sattu” (สะตุ), which is a process of roasting it in a hot pan to obtain a stronger effect(Sommano et al. 2022). In some formulas, cannabis is extracted with oils such as sesame oil and cottonseed oil. In addition, the Department of Traditional and Alternative Medicine (DTAM) in Thailand selected another cannabis oil medicinal formula according to the knowledge of a traditional healer called the Decha formula. It is prepared by boiling and simmering cannabis inflorescences in coconut oil at a ratio of 1:10 while controlling the amount of THC at 2,000 mg/L. The DTAM produced 660,000 bottles for the 22 hospitals, each containing 5 cc of oil, totaling 3,300 L (DTAM 2019). Thai traditional medicine practitioners are recommended for treating patients with insomnia, stress, lack of appetite, migraines, chronic pain, and tremors from Parkinson’s disease.

### The mathematical model of heterogeneous hemp seeds

The heterogeneous hemp seed model demonstrates how optimizing selective roasting methods and solution retention times can effectively extract Δ9-THC within acceptable parameters, thereby reducing excessive removal costs. Additionally, a physical mathematical model for selective chemical removal techniques based on extended diffusion and extraction has been proposed (N.-S. Kim and Han 2006; Kim et al. 2018). In medicine, time-dependent degradation in the gastrointestinal tract is commonly considered, especially for substances with heterogeneous husks and core components. This paper explores the application of a coating to the outer wall to regulate the dissolution rate, allowing for gradual release. Conversely, it introduces a new approach to selectively removing Δ9-THC from hemp seed husks over time, utilizing either physical or chemical methods. A mathematical model is employed for substances with varying chemical concentrations, optimizing the husk removal processes based on physical and chemical dispersion.

The illustration depicts a three-dimensional model of hemp seeds, facilitating the establishment of THC control models through diffusion coefficients. This model enables precise control of the THC content by determining the husk thickness and applying diffusion-based techniques. In the realm of general kinetics, under conditions of mass-transfer control, as described in widely referenced diffusion theory, it is reasonably postulated that the concentration of reactants is directly proportional to the first power of concentration irrespective of temperature(Kim et al. 2018). This phenomenon mirrors the process of product layer control observed in external husks. The successful demonstration of the simulation results highlights the effective application of mathematical models in analyzing natural hemp with husks.

Based on the density-concentration correlation principle, by analyzing the components of husks and soft nut kernels, the Δ9-THC content was integrated using mathematical models to ensure compliance with global regulations. This mathematical model, inspired by traditional Asian medical texts on hemp seed roasting, serves as a stable process for Δ9-THC removal. The findings from the examination of Korean hemp seeds, which revealed a husk Δ9-THC concentration of 300 mg/L and a concentration of less than 2 mg/L in nut kernels, have been reported. These results were subjected to thorough simulation and validation in this study, employing methodologies such as husk thickness determination, theoretical chemical penetration analysis, and dissolution and separation techniques (Aditya et al. 2019).

**Fig. 1:**
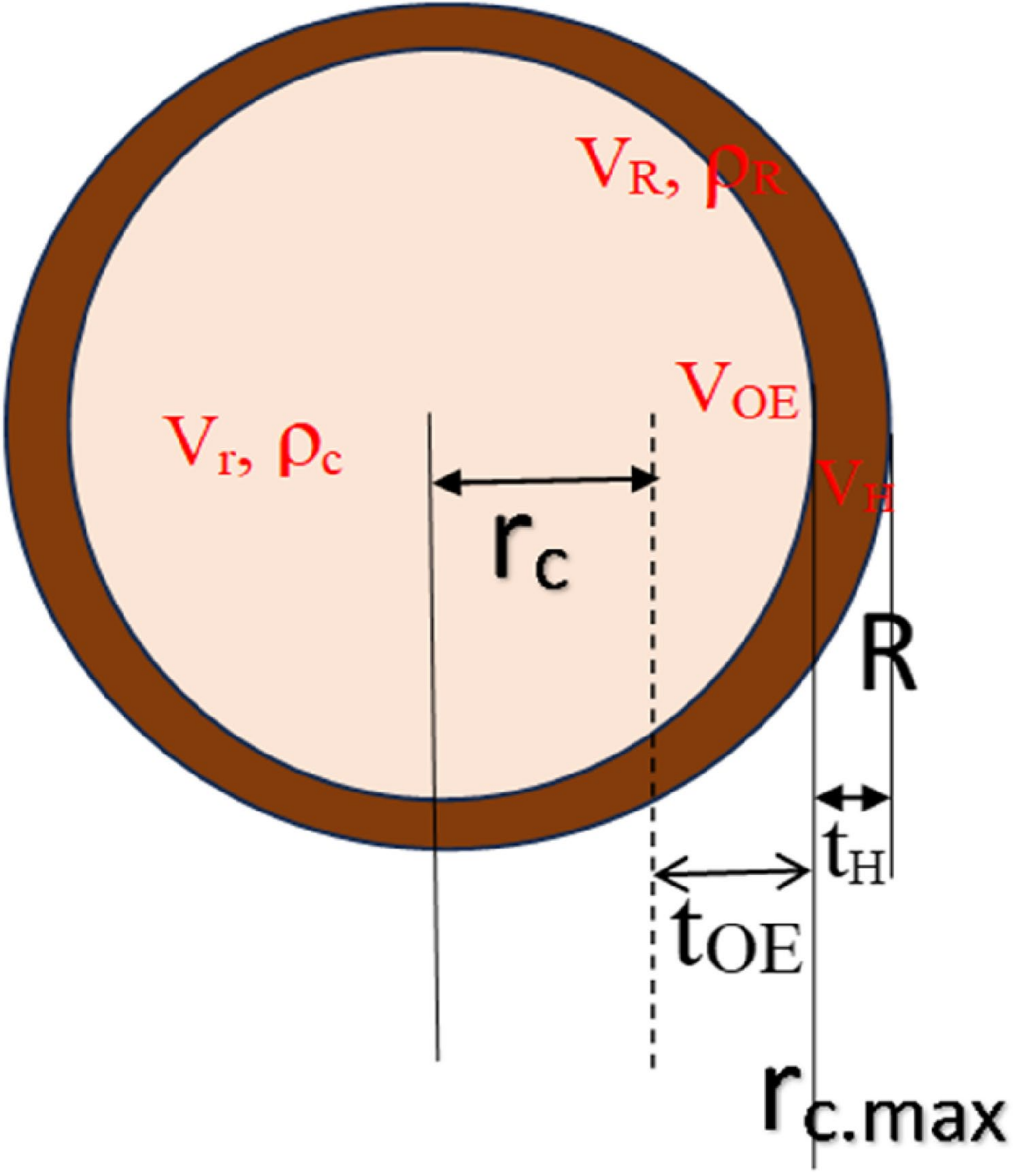
Two-dimensional structure of hemp seed

The two-dimensional structure of the hemp seeds, as shown in Fig. 1, provides an explanation of the removal of the husk, inner components, and areas. The schematic includes the maximum nut kernel radius, *r*_c.max_; the volume, radius, and density of hemp, including the husk and the nut kernels, V_R_, R, and ρ_R_; the volume, radius, and density of the core nut component with the husk removed, V_r_, r, and ρ_c_; the volume, thickness, and density of the husk, V_H_, t_H_, and ρ_H_; and the volume and thickness of the core excessively removed due to processing, V_OE_ and t_OE_. The nut kernel component, with only the husk removed, exhibited a maximum hemp radius _of rc.max_.

### PK-16 analysis and THC-Safe certification

South Korea is among the few countries globally that regulate hemp seed. The permissible THC content in seeds is 5 mg/kg, and in hemp seed oil, it is 10 mg/kg, with similar limits for CBD content. However, revisions to regulations announced on December 29, 2022, prohibit the mention of THC, CBD, or similar substances on labels or in advertising for hemp-related products. Instead, full disclosure of test inspection reports is needed(Hernandez-Ledesma et al., n.d.). Despite stringent regulations, cannabinoid analyses for traditional hemp seeds for food use in Korea have not yet been reported.

Gas chromatography‒mass spectrometry (GC‒MS) and liquid chromatography‒mass spectrometry (LC‒MS) were used for PK-16 cannabis analysis. GC‒MS, which is primarily used for volatile terpenes, separates compounds based on physical and chemical properties and identifies them through mass spectrometry after ionization. LC‒MS, which is widely used in the pharmaceutical and biotechnology industries, separates and quantifies compounds using liquid chromatography. LCMSMS is mainly used for accelerated fragmentation of substances to identify unknown components, while HPLC is primarily used to confirm full potency by comparison with library data. The specific methods and concepts of the PK-16 protocol are detailed in “The Cannabis Story 2” and patented(Kim 2023b). This method was initially applied to husked hemp seeds, and the material density was correlated with the concentration; (Kim 2022)components of the husk and soft core-nut were analyzed using PK-16. Expanding this method to include Δ9-THC content, based on mathematical models, ensures compliance with international regulations.

## Methods

### Analysis procedure using PK-16

Sixteen cannabinoid reference standards were procured from Cayman Chemical, USA, to calibrate the Perkin Elmer LC 300 HPLC-PDA (column: PerkinElmer Quasar SPP C18 Column, 150 × 3.0 mm, 2.6 μm) with Simplicity Chrom software. The reference standards for individual amber glass ampules at a concentration of 1 mg/ml for each cannabinoid included Cannabichromene (CBC), Cannabichromenic acid (CBCA), CBD, CBG, CBN, Cannabidiolic acid (CBDA), Cannabigerolic acid (CBGA), Cannabinolic acid (CBNA), Cannabicyclol (CBL), Cannabidivarin (CBDV), Cannabidivarinic acid (CBDVA), Δ8-Tetrahydrocannabinol (Δ8-THC), Δ9-THC, Δ9-Tetrahydrocannabinolic acid (THCA-A), Tetrahydrocannabivarin (THCV), and Tetrahydrocannabivarinic acid (THCVA).

After the glass ampules containing the reference standards were broken open, they were carefully and swiftly transferred to amber glass vials with airtight caps and refrigerated at −4 °C. HPLC grade solvents such as methanol (MeOH), acetonitrile (ACN), and DI water (H2O), as well as PK0 diluent from Biomedical 3D Printing, were used. The mobile phase and dilutions of the samples were used. Ethanol, MeOH, DI, ACN, PK0 diluent, and interface markers were used in accordance with patent specifications to create the final LC standard solution(Kim 2022).

A stock standard mixture (SSM) with a 50 ppm concentration was prepared by adding 150 µl of each cannabinoid reference standard (a total of 2400 µl) and 600 µl of the mixture. Eight calibration levels with appropriate dilutions were prepared following the preparation of the mobile phases as prescribed by the PK-16 Protocol. Mobile phase A consisted of 1 ml formic acid and 800 µl 10 M ammonium formate in 1 L PK diluent, while mobile phase B consisted of 900 µl formic acid in 900 ml acetonitrile (ACN) along with 100 ml mobile phase A.

An isocratic process with 21.1% mobile phase A and 78.9% mobile phase B was carried out for all the analysis procedures by PK-16. The major difference between PK-16 and PK-16 lies in the pretreatment process and the application of PK0 dilution solution, which is optimized for HPLC equipment and the analysis of 16 types of cannabinoids (PK16 Solution). The analysis protocol used was the same as that used for PK-16, and all the cannabinoids were identified and assigned according to their respective retention times. The samples were analyzed with reference to 16 types of cannabinoid analysis papers. The analysis was conducted at 25 ~ 28 °C with a flow rate of 1.2 mL/min. Ten microliters of the sample was injected, and the sample was analyzed at a wavelength of 220 nm using a PDA detector.

### Hemp oil Preparation

Seven types of natural oils (listed in Table 1) were prepared for the roasting process. Among them, four—canola, palm, soybean, and hemp (imported from Canada)—were purchased as commercially available edible ingredients in quantities ranging from 150 to 250 ml each. The other three types of hemp seeds, namely, HempSeed-1, HempSeed-2, and HempSeed-3 (HSD-1, HSD-2, and HSD-3), were purchased from Andong and Suncheon, Korea. In these three hemp seeds, one-third of the total weight was removed, with more than 99% of the husks removed, and the remaining nut kernels were pressed to obtain THC-Safe certification, ensuring a total THC content of 5 mg/L or less. Fifty milliliters were extracted from each of HSD-1, HSD-2, and HSD-3, totaling 150 ml. This extracted oil was then mixed with Canadian hemp oil (referred to as ‘Hemp’ in Table 1) to prepare a total of 200 ml of hemp oil HSDM (Hemp mixture) for the final roasting process. Deionized (DI) water was primarily used to wash most of the ingredients, and the PK0 to PK-16 protocol was used for diluting the extracted oil or solution. Ethanol diluted to 20% with PK0 solution was used to detect any remaining cannabinoids in the residues.

**Table 1:** Seven different natural oil components prepared for the roasting method **(wt%)**

	Canola (Lin et al. 2013)	Palm (Khosla,n.d.)	Soybean (Ivanov, Lević, and Sredanović, n.d.)	Hemp (Vonapartis et al. 2015)	HSD-1 (Kim 2023b)	HSD-2 (Moon et al. 2006)	HSD-3 (Kim et al. 2023)
Saturated fat	7.4	49.3	15.6	7.0	9.2	8.8	8.0
ω−3 (α-linolenic acid)	9.1	0.2	7.0	22.0	15.1	14.2	14.8
ω−6 (ɣ-linoleic acid)	18.6	9.1	51.0	54.0	47.1	45.1	46.2
ω −9 (Oleic acid)	61.8	40.0	22.6	9.0	10.3	9.9	9.3

### Roasting procedures

The quantity of cannabis seeds and the amount of oil were accurately measured, and the amount of oil obtained after roasting was also precisely measured for analysis. Five grams of cannabis seeds were weighed and used for four types of oils: Canola, Palm, Soybean, and HSDM. Different percentages (10%, 20%, 30%, 40%) of each type of oil were added to the weighed cannabis seeds with husks, and then they were roasted at 50 °C for 30 min to prevent burning. The resulting oil was collected, and its quantity was accurately measured and recorded. The remaining cannabis seeds were thoroughly rinsed with water, equivalent to four times the mass of the cannabis seeds, and then separated. The rinsed seeds were dried, while the water used for rinsing was stored separately. Additionally, to check for residual cannabinoids in the remaining residues, ethanol diluted to 20% with PK0 solution was used.

**Fig. 2:**
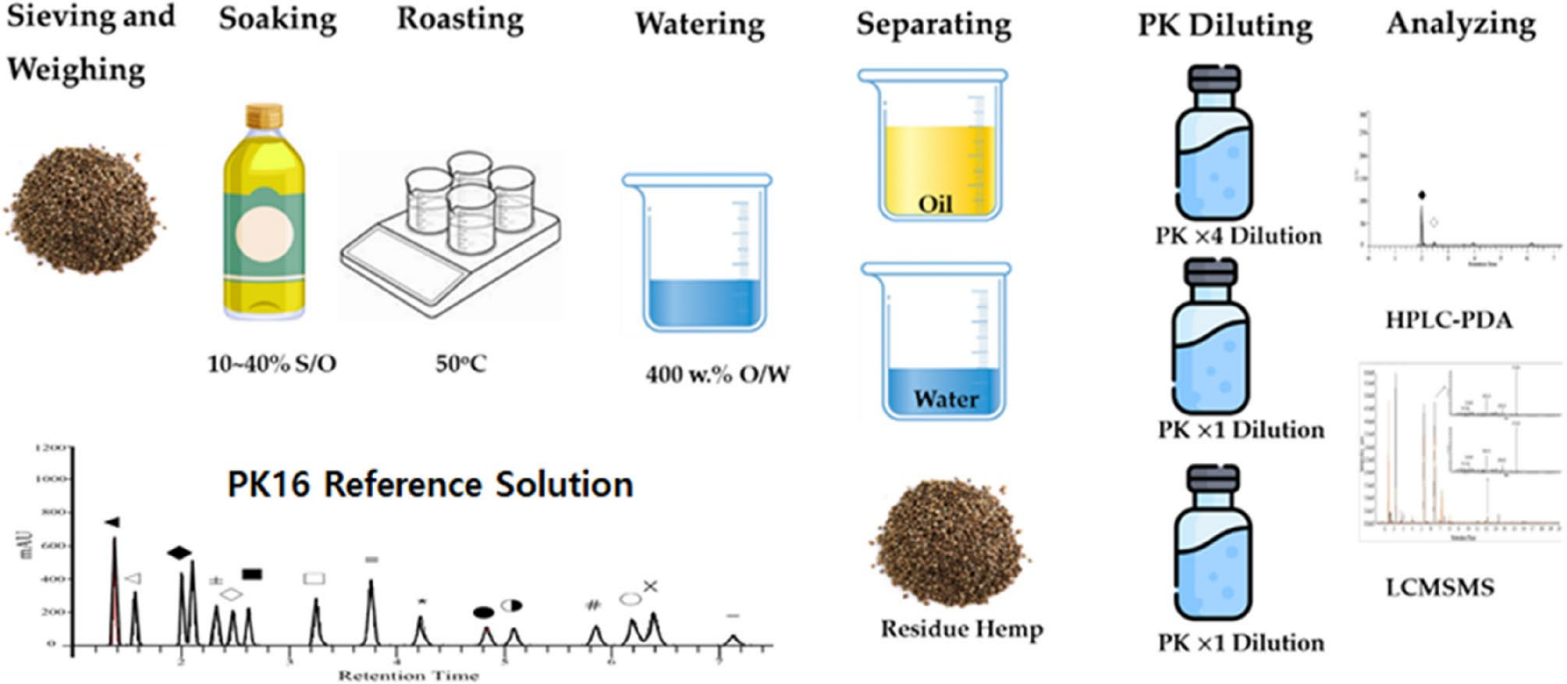
Schematic of the roasting process of hemp by Shengji Zonglu, extraction, separation, and analysis using the PK16 method

Figure 2 shows a schematic of the experiment, where extraction processes based on traditional literature, such as Shengji Zonglu in China’s medical literature, utilize equipment such as a hot plate, balance, beaker, and micropipette (tip). Typically, commercially purchased hemp seeds are taken in 5 g increments, mixed with oil at ratios ranging from 10% to 40% by weight, and roasted at 50 °C for 30 min. Subsequently, they are diluted with deionized water at a ratio of four times the oil volume to prevent surface burning of the hemp seeds. The oil, water, and residual hemp seeds are then separated and stored. Each separated component was diluted with PK0 at ratios of 4:1, 1:1, and 1:1 and then analyzed using LC-MS/MS and HPLC. The PK-16 analysis protocol used was compared to the PK16 Reference Solution library for analysis.

## Results and discussion

### Mathematical model simulation and analysis

The practice of applying a thin coating to oral medications to regulate their dissolution in the human gastrointestinal tract over time is a common technique in the pharmaceutical industry. A mathematical model is utilized for substances with varying chemical concentrations to optimize husk removal processes through physical and chemical dispersion. From the perspective of physical coating and mechanical separation, the PK research group was able to establish a mathematical model of natural hemp seed, allowing us to estimate changes in husk thickness and density through empirical experiments. Building upon this theoretical foundation, the PK research group proposed a mathematical model for the safe removal of Δ9-THC from natural hemp by extending the concept of density, a physical attribute of nut kernels and husks, to Δ9-THC, a component significantly different from other materials. This provides a practical and effective method for controlling the total Δ9-THC content in various pharmaceuticals or healthy foods worldwide through the safe and practical chemical extraction of Δ9-THC. This method enables rapid, economical, and efficient control of the Δ9-THC content in hemp seeds without the need for physical removal.

In general, when all the husks are removed and only a portion of the core nuts remains, the application of the following mathematical model is possible.

$$\:{\text{V}}_{\text{r}\text{e}\text{d}}\equiv\:\:{\text{V}}_{\text{R}}-{\text{V}}_{\text{r}}$$

When $$\:\:0\le\:{\text{r}}_{\text{c}}\le\:{\text{r}}_{\text{c}.\text{m}\text{a}\text{x}}\:$$

$$\:{\text{V}}_{\text{r}\text{e}\text{d}}=4{\uppi\:}{\int\:}_{{\text{r}}_{\text{c}}}^{\text{R}}{\text{r}}^{2}\text{d}\text{r}\:\text{a}\text{n}\text{d}\:{{\uprho\:}}_{\text{c}}\equiv\:\:\frac{{\text{W}}_{\text{c}}}{{\text{V}}_{\text{c}}}\:$$

$$\:{\text{V}}_{\text{r}\text{e}\text{d}}=\:{\text{V}}_{\text{H}}+{\text{V}}_{\text{O}\text{E}}\:\text{a}\text{n}\text{d}{\text{t}}_{\text{O}\text{E}}=\text{R}-\:{\text{r}}_{\text{c}}-{\text{t}}_{\text{H}}$$

$$\:{\text{V}}_{\text{r}\text{e}\text{m}\text{a}\text{i}\text{n}}\equiv\:\:{\text{V}}_{\text{R}}-{\text{V}}_{\text{r}\text{e}\text{d}}={\text{V}}_{\text{R}}-\left({\text{V}}_{\text{R}}-{\text{V}}_{\text{r}}\right)={\text{V}}_{\text{r}}$$.

Checking the given equation, we observe that $\:0\le\:{\text{t}}_{\text{O}\text{E}}\le\:{\text{r}}_{\text{c}.\text{m}\text{a}\text{x}}$, and under the condition where $\:{\text{r}}_{\text{C}}={\text{r}}_{\text{c}.\text{m}\text{a}\text{x}}$, $\:\text{R}=\:{\text{r}}_{\text{c}.\text{m}\text{a}\text{x}}+{\text{t}}_{\text{H}}$, making $\:{\text{t}}_{\text{O}\text{E}}=0$. This signifies a state where only the husk is perfectly removed in the process. Another extreme condition is when $\:{\text{r}}_{\text{C}}=0$, where $\:{\text{t}}_{\text{O}\text{E}}=\text{R}-{\text{t}}_{\text{H}}$=$\:{\text{r}}_{\text{c}.\text{m}\text{a}\text{x}}$. This implies removing all core nuts, including the husk, leaving nothing behind. The theoretical goal is to achieve a successful process by minimizing t_OE_, the removal of husk only. In this case, the density of the residue after the process is determined by $\:{{\uprho\:}}_{\text{R}\text{e}\text{m}\text{a}\text{i}\text{n}}\equiv\:\:\frac{{\text{W}}_{\text{R}\text{e}\text{m}\text{a}\text{i}\text{n}}}{{\text{V}}_{\text{R}\text{e}\text{m}\text{a}\text{i}\text{n}}}$, where applying $\:{\text{W}}_{\text{r}\text{e}\text{m}\text{a}\text{i}\text{n}}=\:{{{\uprho\:}}_{\text{c}}\text{V}}_{\text{r}}$ yields a final material density of $\:{{\uprho\:}}_{\text{R}\text{e}\text{m}\text{a}\text{i}\text{n}}=\:\frac{{{{\uprho\:}}_{\text{c}}\text{V}}_{\text{r}}}{{\text{V}}_{\text{r}}}={{\uprho\:}}_{\text{c}}$.

In general, when not all the husks are removed and some core nuts are left intact along with some portion of the husk, the following mathematical model can be applied.

$$\:{\text{V}}_{\text{r}\text{e}\text{d}}\equiv\:\:{\text{V}}_{\text{R}}-{\text{V}}_{\text{r}}$$

When $$\:\:{\text{r}}_{\text{c}.\text{m}\text{a}\text{x}}\le\:{\text{r}}_{\text{c}}\le\:\:\text{R}$$

$$\:{\text{V}}_{\text{r}\text{e}\text{d}}=4{\uppi\:}{\int\:}_{{\text{r}}_{\text{c}}}^{\text{R}}{\text{r}}^{2}\text{d}\text{r}\:\text{a}\text{n}\text{d}\:{{\uprho\:}}_{\text{c}}\equiv\:\:\frac{{\text{W}}_{\text{c}}}{{\text{V}}_{\text{c}}}\:$$.

Under the above conditions, hemp retains all the core nuts, leaving some of the shells intact. This can be represented in terms of volume as follows:

$\:{\text{V}}_{\text{r}\text{e}\text{m}\text{a}\text{i}\text{n}}\equiv\:\:{\text{V}}_{\text{r}}$ When $\:\:{\text{r}}_{\text{c}.\text{m}\text{a}\text{x}}\le\:{\text{r}}_{\text{c}}\le\:\:\text{R}$, in this case, the remaining hemp consists of both core nuts and some portion of the shells. We can further divide this process as follows: under these conditions, two different components of materials exist.

$$\begin{aligned}&\:{\text{V}}_{\text{r}\text{e}\text{m}\text{a}\text{i}\text{n}}={\text{V}}_{\text{C}.\text{m}\text{a}\text{x}}+{(\text{V}}_{\text{H}}-{\text{V}}_{\text{R}}+{\text{V}}_{\text{r}});\\&:{{\uprho\:}}_{\text{c}}\equiv\:\:\frac{{\text{W}}_{\text{c}}}{{\text{V}}_{\text{c}}}\:\text{a}\text{n}\text{d}{{\uprho\:}}_{\text{H}}\equiv\:\:\frac{{\text{W}}_{\text{H}}}{{\text{V}}_{\text{H}}}\end{aligned}$$

$$\begin{aligned}&\:{\text{W}}_{\text{r}\text{e}\text{m}\text{a}\text{i}\text{n}}\equiv\:\:{{{\uprho\:}}_{\text{c}}\text{V}}_{\text{C}.\text{m}\text{a}\text{x}}+{{\uprho\:}}_{\text{H}}{(\text{V}}_{\text{H}}-{\text{V}}_{\text{R}}+{\text{V}}_{\text{r}});\\&:{{\uprho\:}}_{\text{c}}\equiv\:\:\frac{{\text{W}}_{\text{c}}}{{\text{V}}_{\text{c}}}\:\text{a}\text{n}\text{d}{{\uprho\:}}_{\text{H}}\equiv\:\:\frac{{\text{W}}_{\text{H}}}{{\text{V}}_{\text{H}}}\end{aligned}$$

*Here*, $\:{\text{V}}_{\text{H}}={\text{V}}_{\text{R}}-{\text{V}}_{\text{C}.\text{m}\text{a}\text{x}}$ if we rearrange the equations above,

$$\begin{aligned}\:{\text{W}}_{\text{r}\text{e}\text{m}\text{a}\text{i}\text{n}}&\equiv\:\:{{{\uprho\:}}_{\text{c}}\text{V}}_{\text{C}.\text{m}\text{a}\text{x}}+{{\uprho\:}}_{\text{H}}({\text{V}}_{\text{r}}-{\text{V}}_{C.Max})\\&=\:{{\uprho\:}}_{\text{H}}{\text{V}}_{\text{r}}-({{\uprho\:}}_{\text{H}}-{{\uprho\:}}_{\text{c}}){\text{V}}_{C.Max}\end{aligned}$$

$$\:{{\uprho\:}}_{\text{R}\text{e}\text{m}\text{a}\text{i}\text{n}}\equiv\:\:\frac{{\text{W}}_{\text{R}\text{e}\text{m}\text{a}\text{i}\text{n}}}{{\text{V}}_{\text{R}\text{e}\text{m}\text{a}\text{i}\text{n}}}$$

$$\begin{aligned}\:{{\uprho\:}}_{\text{R}\text{e}\text{m}\text{a}\text{i}\text{n}}&=\:\frac{{{\uprho\:}}_{\text{H}}{\text{V}}_{\text{r}}-({{\uprho\:}}_{\text{H}}-{{\uprho\:}}_{\text{c}}){\text{V}}_{C.Max}}{{\text{V}}_{\text{r}}}\\&={{\uprho\:}}_{\text{H}}-({{\uprho\:}}_{\text{H}}-{{\uprho\:}}_{\text{c}})\frac{{\text{V}}_{C.Max}}{{\text{V}}_{\text{r}}}\end{aligned}$$

When the husk of the material is completely removed, $\:{\text{V}}_{\text{r}}\approx\:{\text{V}}_{C.Max}$, and $\:{{\uprho\:}}_{\text{R}\text{e}\text{m}\text{a}\text{i}\text{n}}\approx\:\:{{\uprho\:}}_{\text{c}}$. However, if the husk of the hemp is not properly removed, then $\:{{\uprho\:}}_{\text{R}\text{e}\text{m}\text{a}\text{i}\text{n}}={{\uprho\:}}_{\text{H}}-({{\uprho\:}}_{\text{H}}-{{\uprho\:}}_{\text{c}})\frac{{\text{V}}_{C.Max}}{{\text{V}}_{\text{r}}}$. Assuming a general scenario where the volume of the husk is assumed to be 10% of the hemp and the density of the husk is approximately 1.5 times the density of the nut kernels ($\:{{\uprho\:}}_{\text{H}}=1.5{{\uprho\:}}_{\text{c}}$), the final density of the material without removing the husk is $\:{{\uprho\:}}_{\text{R}\text{e}\text{m}\text{a}\text{i}\text{n}}=1.05{{\uprho\:}}_{\text{c}}$. Hence, depending on the extent of husk removal, the final material density falls within this range. $\:{{\uprho\:}}_{\text{c}}\le\:{{\uprho\:}}_{\text{R}\text{e}\text{m}\text{a}\text{i}\text{n}}\le\:1.05{{\uprho\:}}_{\text{c}}$.

Eventually, the results obtained through the analysis of physical models can be extended as follows. Assuming compatibility between the units of density and concentration, we use the unit of mg/L collectively. By expanding the concept under this assumption, we can rewrite it as $\:\:{{\uprho\:}}_{\text{H}.\text{T}\text{H}\text{C}}\cong\:{\text{C}}_{\text{H}.\text{T}\text{H}\text{C}}$and summarize it as follows.

$$\begin{aligned}\:{C}_{\text{R}\text{e}\text{m}\text{a}\text{i}\text{n}.\text{T}\text{H}\text{C}}&=\:\frac{{{\uprho\:}}_{\text{H}}{\text{V}}_{\text{r}}-({{\uprho\:}}_{\text{H}}-{{\uprho\:}}_{\text{c}}){\text{V}}_{C.Max}}{{\text{V}}_{\text{r}}}\\&\cong\:{\text{C}}_{\text{H}.\text{T}\text{H}\text{C}}-\left({\text{C}}_{\text{H}.\text{T}\text{H}\text{C}}-{\text{C}}_{\text{C}.\text{T}\text{H}\text{C}}\right)\frac{{\text{V}}_{C.Max}}{{\text{V}}_{\text{r}}}\end{aligned}$$

$$\begin{aligned}\:{C}_{\text{R}\text{e}\text{m}\text{a}\text{i}\text{n}.\text{T}\text{H}\text{C}}&={\text{C}}_{\text{H}.\text{T}\text{H}\text{C}}-\left({\text{C}}_{\text{H}.\text{T}\text{H}\text{C}}-{\text{C}}_{\text{C}.\text{T}\text{H}\text{C}}\right)\frac{{\text{V}}_{\text{R}}{-\text{V}}_{H}}{{\text{V}}_{\text{R}}{-\text{V}}_{H}-{\text{V}}_{\text{O}\text{E}}}\\&={\text{C}}_{\text{H}.\text{T}\text{H}\text{C}}-({\text{C}}_{\text{H}.\text{T}\text{H}\text{C}}-{\text{C}}_{\text{C}.\text{T}\text{H}\text{C}})\frac{{\text{V}}_{\text{R}}{-\text{V}}_{H}}{{\text{V}}_{\text{R}}-x\left({\text{V}}_{\text{R}}\right)}\end{aligned}$$

**Fig. 3:**
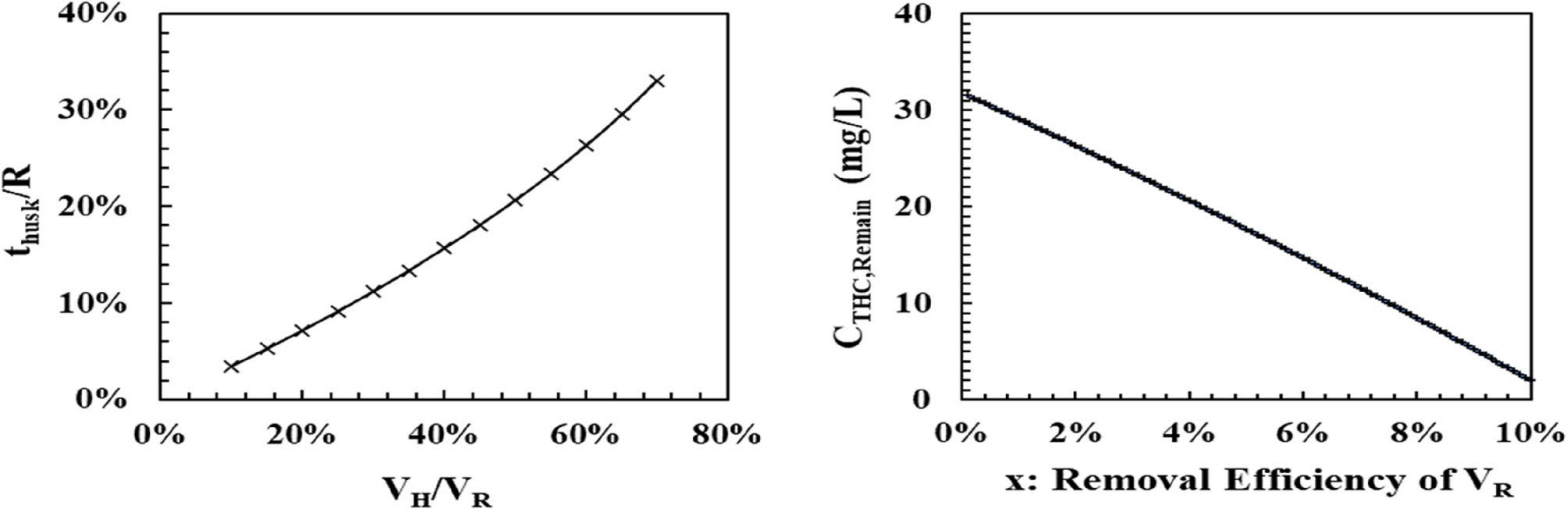
Simulation results, based on the mathematical model for hemp and processing procedures, are as follows: Left: Simulation results for hemp size and husk thickness before processing, according to the ratio of husk volume to the total hemp volume. Right: Assuming that the husk constitutes 10% of the total volume, the simulation results show the change in the THC content of the remaining material when the husk is removed from 0% to 10% (assumed value)

$\:{C}_{\text{R}\text{e}\text{m}\text{a}\text{i}\text{n}.\text{T}\text{H}\text{C}}={\text{C}}_{\text{H}.\text{T}\text{H}\text{C}}-({\text{C}}_{\text{H}.\text{T}\text{H}\text{C}}-{\text{C}}_{\text{C}.\text{T}\text{H}\text{C}})\frac{(1-\frac{{\text{V}}_{H}}{{\text{V}}_{\text{R}}})}{(1-x)}$. If we assume that the removed fractional volume is x,

Analyzing the equation based on field data and density, assuming that x is 40–60% or that at least one-third of the weight or volume of the material is removed, we obtain the following result in Fig. 3. Based on the analysis using the figures below, simulations were conducted for the husk volume relative to the total volume of hemp seeds (V_H_/V_R_), ranging from 10% to 75%. According to the results, the husk size ranges from 2% to 33% relative to the radius (t_husk_/R). This can be verified by examining Fig. 3-left of the simulation results below. Another aspect to consider is whether our current practice of physically removing more than one-third or practically 40–60% is appropriate. From both the physics-based simulations and experimental results, we assume that V_H_ occupies 10% of the total volume (V_R_), and based on this assumption, we detail the results assuming a removal of 0–10%, as depicted in Fig. 3-right. According to Korean standards, removing more than 9.1% of V_H_ would result in a product that meets the current criteria.

### Hempseeds analysis

**Fig. 4:**
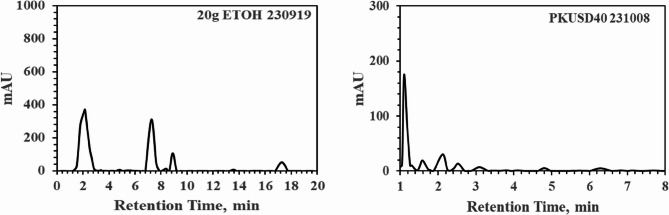
Graphical data obtained from HPLC-PDA (PerkinElmer 300 series) at 220 nm, utilizing methanol for the extraction of HSD-1. Left: AOA under operating conditions. Right: PK-16 protocol under operating conditions

The following experiment involved extracting 20 g of HSD-1 from the entire husk of Andong, Koea, for 2 h using 20% methanol (Sigma‒Aldrich Inc.), a commonly used method for hemp extraction, and the results are presented in the Fig. below. Although the analysis method shown in Fig. 4-left was performed using HPLC conditions optimized for the PK-16 method, it was not fully optimized for AOA(Vaclavik et al. 2019). However, as observed, peaks were detected at approximately 7–8 min, near 8 and 9 min, and at 13 and 17 min, respectively. Upon further analysis using LC‒MS/MS, it was determined that the peak at approximately 7 min was related to Δ9-THC, indicating that the analyzed hemp seeds contained a significant number of husks containing more than 50 mg/L Δ9-THC. Upon closer examination, it was evident that peaks did not appear until approximately 20 min, indicating that the method was not optimized. To address this, faster and more accurate data analysis is needed, focusing on lower concentrations and selecting all peaks within 10 min. Moreover, the general experimental results revealed that various cannabinoids, including CBD and Δ9-THC, were present in the husks.

As shown in Fig. 4-right, these results were obtained by employing a similar methodology to that of Fig. left, utilizing 20% methanol containing the entire shell for extraction over 2 h, followed by analysis according to the PK-16 protocol. By precisely adjusting the quantities of A and B in the mobile phase or the quantity of PK0, hydrophobic cannabinoids can be optimized to appear rapidly or slowly, with the aim of no peaks appearing ideally even by 8 min postinjection. The primary objective depicted in Fig. 4 right below was to ensure that all analyzable cannabinoids passed through the column without any peaks appearing within 10 min. We also confirmed through LC-MS/MS that the peak appearing at approximately 5 min is associated with Δ9-THC. Such stable and rapid analytical techniques necessitate numerous experiments and repetitions that need to be optimized by analyzing post-roasting, rinsing solutions and residues for input into the library.

**Fig. 5:**
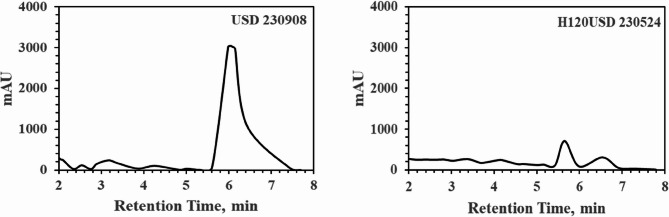
Graphical data obtained from HPLC-PDA (PerkinElmer 300 series) at 220 nm, utilizing methanol for the extraction of separated husks from HSD-2. Left: AOA under operating conditions. Right: samples were extracted with hot water and analyzed using the PK-16 protocol under operating conditions

The chemical analysis experiments primarily focused on nut kernels that had been physically separated and whose husks had been removed. In the following experiment, 5 g of HSD-2 was extracted from Suncheon, Korea, as shown in the results below, using 20% methanol for a 30-minute extraction process, including the entire husk. This extract was analyzed using the AOA method, and the results are presented in Fig. 5 below. By comparing the results obtained using the same method to those presented in Fig. 4-left, where both the nut kernels and husks of the seeds were analyzed, we aimed to confirm the high absorbance between retention times 6 and 8 on the husks. In the husk portion, the Δ9-THC content exceeded that of the nut kernels by more than 100 times.

In this manner, we heated water for 30 min on a hot plate with a temperature exceeding 120 °C and extracted it using PK-16, and the results are displayed in Fig. 5-right. In this case, when analyzing PK-16, the Δ9-THC peak appears at approximately 5 to 6 min, with an increase in the amounts of methanol and PK0 causing the peak to shift to the right. Compared with Fig. 4-right above, we observe significantly more than 60 times more Δ9-THC extracted from the separated husk, indicating that effective extraction is ultimately possible using high-temperature water and a slight addition of ethyl alcohol (methyl alcohol in the present paper). However, minimizing the use of high-temperature processes and alcohols such as methyl alcohol and using pure oil for extraction may offer new possibilities in terms of economy, stability, and safety.

Utilizing the solubility of THC and CBD to assess their hydrophilicity and lipophilicity is crucial. The selective dissolution of cannabinoids is a valuable technique, particularly in sample preparation and analysis, for which PK0 is used to remove impurities with a retention time of approximately 1 min. CBD exhibits low water solubility (12.6 mg/L) and high lipophilicity (logP of 6.3), indicating a preference for fats over water. Additionally, it demonstrates weakly acidic properties with a pKa of 9.1, a melting point of 67 °C, and a molar mass of 314 g/mol. Ethanol, methanol, DMSO, and dimethyl formamide, purged with inert gas, are suitable solvents for CBD dissolution, with approximate solubilities in these solvents of approximately 35, 30, 60, and 50 mg/mL, respectively. In contrast, THC exhibits very low solubility in water, measured at 0.0028 mg/mL. The experimental focus shifts from AOA to PK-16 with the aim of reducing the retention time and enhancing reliability without relying excessively on reference solutions. In contrast to previous research that concentrated on physically removing husks, this study emphasized chemical extraction, encompassing husks, and employing diffusion models in geometric models. PK-16 technology involves controlling the solubility of cannabinoids in hydrophilic parts, which are typically overlooked among the hundreds of cannabinoids present in hemp, instead of increasing their solubility in methanol or lipophilic parts. This method improves solubility in hydrophilic portions, ensuring rapid dissolution of impurities in the area of interest, thereby facilitating effective separation.

**Fig. 6:**
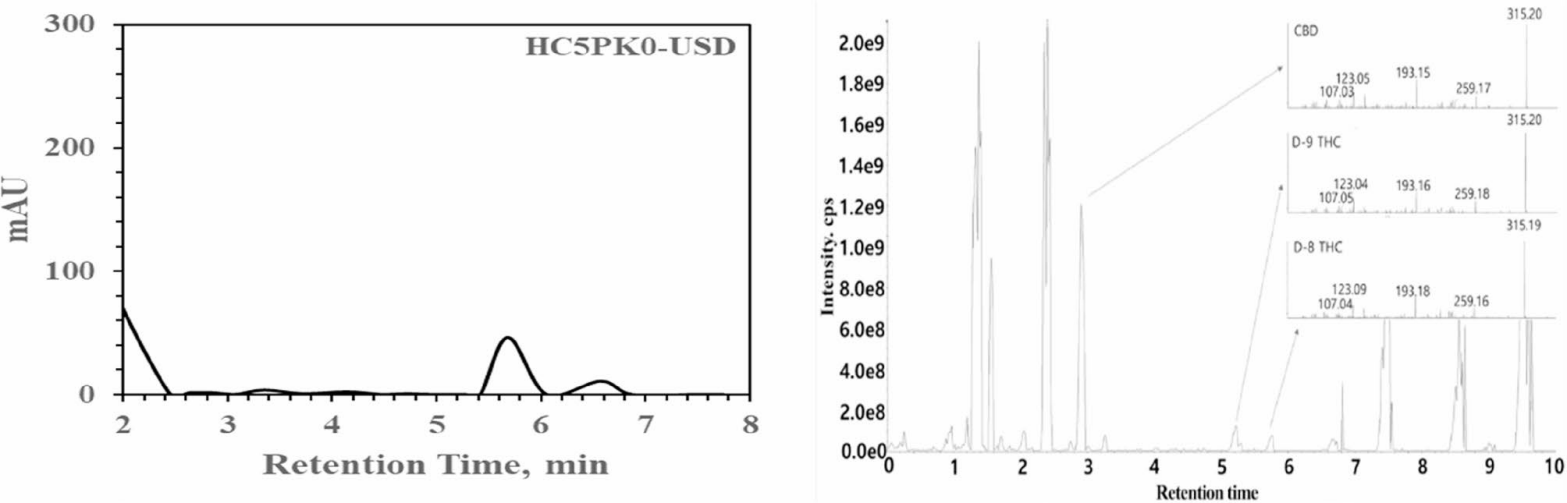
Graphical data were obtained from HPLC-PDA (PerkinElmer 300 series) at 220 nm and LCMSMS (SCIEX 5500) utilizing PK0 for the extraction of HSD-3. On the left, HPLC potency data for HSD-3 are presented, while on the right, LC-MS/MS data for HSD-3 obtained by the PK-16 protocol are shown

This change in methodology has been validated through multiple experiments to ensure economically viable and reliable analysis. Typically, HPLC peaks associated with CBD vary depending on the conditions, but they generally appear at approximately 3 min of retention time in the PK protocol, as shown in Fig. 6-left. Peaks related to THC, on the other hand, tended to emerge between 5 and 6 min. Ultimately, to confirm this component, it was verified using LC-MS/MS, and detailed explanations of the THC-related peaks are provided in Fig. 6-right, based on a thorough examination of the molecular weight of 314 g/mol and fragmented pieces of 193 and 259 g/mol. These experiments confirmed the significance of peak analysis centered at approximately 4 to 6 min, where peaks appeared in the roasting oil, PK solution, and residues based on PK-16 analysis.

### Hempseed roasting

**Fig. 7:**
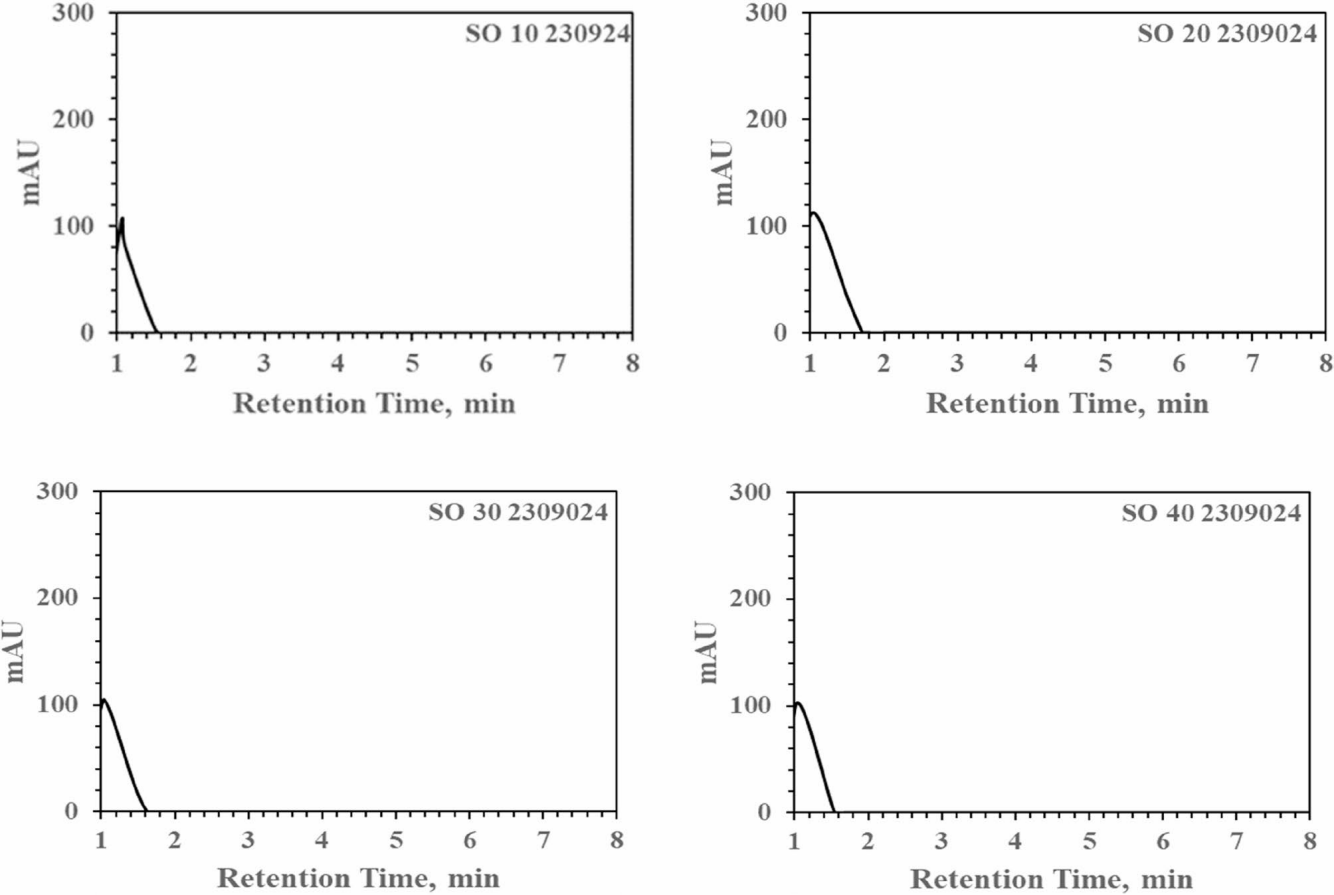
Graphical data obtained by operating HPLC-PDA (PerkinElmer 300 series) at 220 nm using PK0 showing increasing ratios of hemp seeds to oil at 10%, 20%, 30%, and 40%

Roasting was conducted on 5 g of HSD-1, HSD-2, and HSD-3, along with hemp seeds, using four types of oils—Canola, Palm, Soybean, and a hemp mixture (HSDM)—at 50 °C for 30 min to conduct solvent extraction. Increasing the oil ratio from 10% to 40% resulted in similar extraction patterns regardless of the oil type; however, below 20%, insufficient oil separation occurred due to significant absorption by the hemp seeds. Adequate oil separation for analysis was ensured by employing conditions exceeding 30% oil content to solid hemp seeds. In Fig. 7 below, which includes four graphs, hemp seeds roasted in soybean oil were analyzed using PK-16. The effective separation of cannabinoids from oil was not achieved within the roasted oil, with predominantly lipophilic oils separating within 1.5 min of retention, indicating high peaks. Considering subsequent washing processes involving water, hemp oil predominantly underwent simple gravity separation, showing minimal changes in extraction amounts with increasing oil quantity, as confirmed in Fig. 7. Upon confirmation, no peaks related to cannabinoids were observed in the spectra of the separated oils after 2 min. From a THC perspective, this solution can be safely used in food or medical products. Although temperature-related degradation was experimentally investigated and 50 °C for 30 min was confirmed to be safe, detailed explanations are not provided because the focus was not on THC-related aspects.

### Hempseed washing

Another important aspect of this experiment is the issue of field applicability to the process. To minimize significant changes in the analysis results by adjusting the solubility and hydrophilicity of the cannabinoids, a simple yet effective washing method is necessary. If a gravity solid-liquid separation method is applied without applying pressure or using special methods within the hemp after oil roasting, 5–25% of the oil used for roasting remains absorbed or adsorbed without being separated. Processes involving pressing or other methods may incur costs due to additional loss of important components or uneconomical methods, thus posing barriers to commercialization. It is necessary to attempt repetitive washing processes at room temperature (25 °C) with minimal washing solution.

**Fig. 8:**
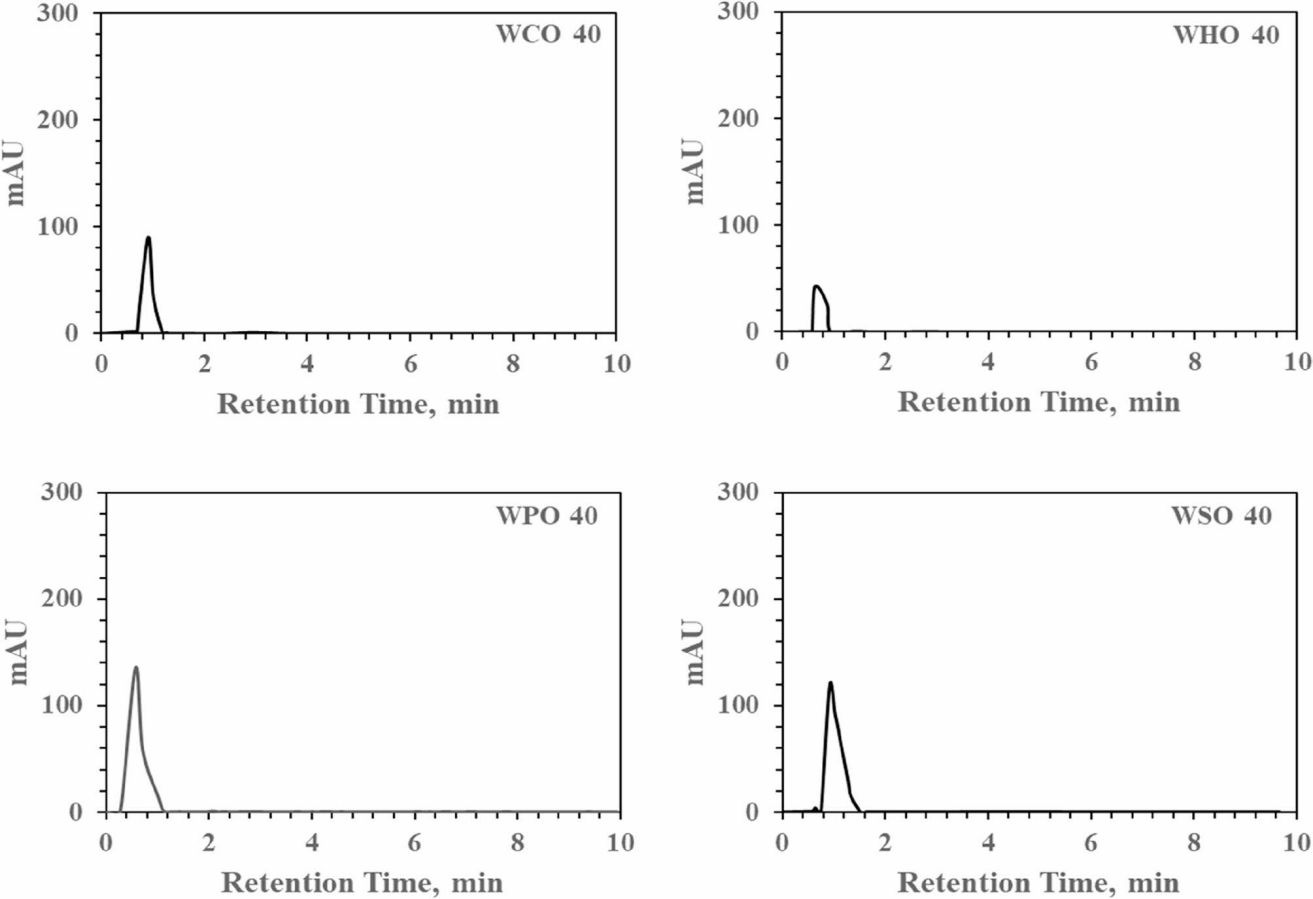
Graphical data obtained from HPLC-PDA (PerkinElmer 300 series) at 220 nm using washing water from four types of 40% oils: Canola, HSDM, Palm, and soybean

In this study, oil and washing water were not mixed, and the various components of cannabinoids, which are soluble in water, were utilized to physically separate residual water-soluble components and oil by repetitively reusing approximately four times the volume of room temperature water for washing. The results are depicted in four graphs in Fig. 8. The analysis was performed using HPLC conditions optimized for the PK-16 method. This process confirmed the reusability of the washing solution for materials for future commercialization. By repeatedly using the same amount of washing water for the same oils at roasting rates of 10%, 20%, 30%, and 40%, while supplementing only 1.3–5.2 ml of water to make the final washing solution 20 ml and using only 1 ml of the separated solution for analysis, similar results were obtained after up to four wash cycles. Figure 8 illustrates the final result of repeatedly using the washing solution with 40% oil. After analysis and roasting, absorption, and adsorption, 20 ml of washing water was added, and the oil and water were separated by gravity in an ultrasonic mixer for 5 min. As shown in Fig. 8, regardless of the type, most of the oil- and water-soluble components were separated within the first 2 min when water was used. Although the amount of ingredients with a retention time of less than 2 min was relatively lower when using HSDM, similar phenomena were observed regardless of the type of oil in terms of absolute quantity.

### Residue analysis

The primary aim of this study was to ensure the production of safe products (residues) by analyzing the amount of cannabinoids in the residue. While this paper emphasizes the residue, in practical industry settings, the residue serves as a foundational material for final processed products, such as being pressed into oil or dried into cereal. Therefore, obtaining THC-safe certification through PK-16 analysis is the ultimate objective. Exploring novel possibilities, beginning from roasting hemp seeds, as documented in ancient literature, and examining extraction methods such as oil, water, and alcohol, constitute another crucial aspect of this study. Despite comparisons to the emerging narcotics crisis, hemp-based technologies have been utilized by communities for millennia. Specifically, it has been confirmed that when THC is managed appropriately to control residues, the application of hemp for food and medical purposes is positively evaluated.

**Fig. 9:**
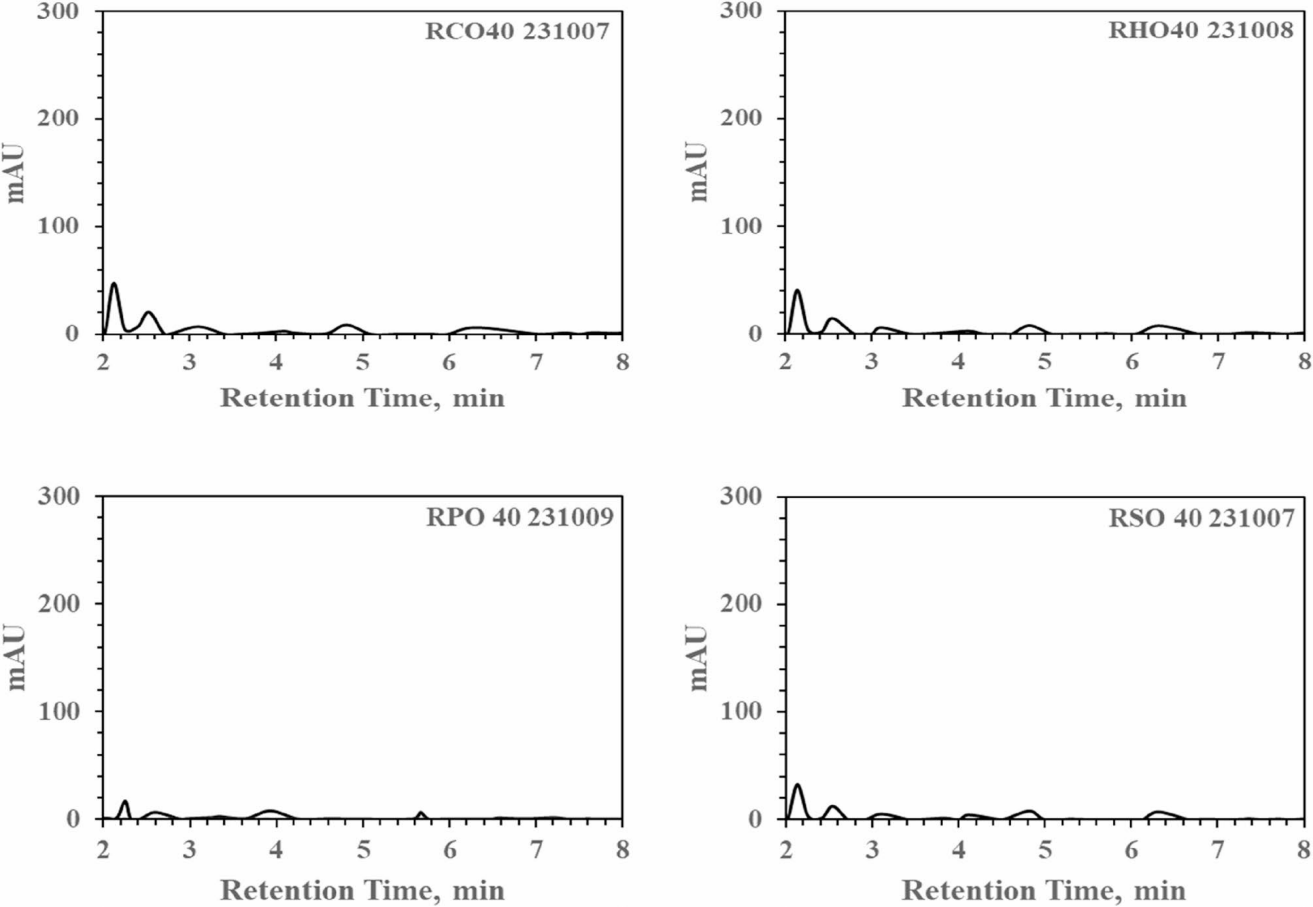
Graphical data obtained from HPLC-PDA (PerkinElmer 300 series) at 220 nm using the

Additionally, providing guidelines for the safe, easy, and stable application of Δ9-THC in different countries’ standards from the final cannabinoids, beyond the field applicability and economic feasibility of the process, was crucial. This method, based on technology patented in 2023, was used with limited data approval. The final step for THC-Safe certification involves GMP-level process management to ensure that the standards are not exceeded in both the solutions and products used in the process and the use of ISO 19,025 (KOLAS in the case of Korea) analytical techniques. Through this process, the Δ9-THC present in the husks was directly extracted and selectively removed under optimal conditions by controlling the diffusion rate, concentration, and temperature of the oil. Extraction was carried out at 50 °C for 30 min regardless of the type of oil, and the residue product obtained a THC-safe stabilization level suitable for medical, edible, and cosmetic use by adding small amounts of the patented ingredients for residual safety. After the process, the residue was ground and sieved to below 200 microns, extracted using PK0, and analyzed using PK-16 HPLC. As a result, it was confirmed that when compounds related to Δ8-THC or Δ9-THC, which are presumed to be between 4 and 6 min in Fig. 9, are combined, they do not exceed 1.5–3.3 mg/kg in total, complying with Korea’s strictest regulations.

residues from four types of 40% oils: Canola, HSDM, Palm, and Soybean.

## Conclusion

The utilization of industrial hemp for medical purposes has faced global challenges due to the lack of integration between regulatory science and extraction, analysis, and clinical techniques. In contrast, this technology provides extraction and analysis methods that strictly adhere to rigorous regulations, minimizing the loss of valuable hemp components by scientifically analyzing hemp based on mathematical and chemical diffusion models to control diffusion rates according to its thickness and diffusivity. Various cannabinoids, including CBD and Δ9-THC, were detected in hemp seed husks, and their selective removal has become a groundbreaking and commercially viable guideline. Chemical extraction techniques using oil roasting and ethanol, based on differences in cannabinoid solubility, were employed to control cannabinoids within hemp seeds. THC removal was confirmed through repeated experiments and residual THC content analysis. PK-16 analysis revealed a THC content of 300 mg/kg in hemp seeds and 2 mg/kg in nut kernels. This selective THC removal method, without physical elimination, is a simple, efficient, and economically feasible approach for future hemp seed oil production and analysis.

## Data Availability

Not Applicable.
